# Elaboration of geopolymer binders from poor kaolin and dam sludge waste

**DOI:** 10.1016/j.heliyon.2019.e01938

**Published:** 2019-06-17

**Authors:** Meriem Merabtene, Larbi Kacimi, Pierre Clastres

**Affiliations:** a Laboratoire des Eco-Matériaux Fonctionnels et Nanostructurés (LEMFN), Université des Sciences et de la Technologie d’Oran- Mohamed Boudiaf, B.P. 1505 El Mnouar, USTO, Oran, Algeria; b Laboratoire Matériaux et Durabilité des Constructions (LMDC), INSA-UPS, Toulouse, France

**Keywords:** Environmental science, Geochemistry, Geopolymer, Alkaline solution, Dam sludge, Kaolin, Binder

## Abstract

The aim of this study is to synthesize geopolymer binders as an ecological alternative for conventional cement. It is intended to have geopolymer binders with molecular composition close to (4SiO_2_.Al_2_O_3_.K_2_O). They were obtained from Algerian poor natural kaolin and dam sludge after calcination at 800 °C. The mixture was attacked by KOH-alkaline solution of 8M and dried in an oven to 40 °C. Fluorescence X analysis, X-ray Diffraction, Thermal Analysis, Infrared analysis, BET technique and Scanning Electronic Microscopy were used to characterize the synthesized geopolymer. Mechanical tests of pure paste were carried out on cylindrical specimens of 20mm diameter and 20mm height to evaluate the mechanical performance of these geopolymers. The results showed that the elaborated products were composed from an amorphous phase of geopolymer molecules with other crystallized phases of aluminosilicates. The infrared analysis revealed functional groups of (–Si-O-Al- and -Si-O-Si-), which could testify to the formation of geopolymer binders. The mechanical test results indicated that these geopolymers have a compressive strength of over 33 MPa after 28 days of hardening.

## 1 Introduction

For several decades, research on economic and ecological hydraulic binders has become a major concern. In material science new binders known as "geopolymers" have been developed over the last years for different application areas such as civil engineering, construction and building restoration.

Geopolymer binders are considered as an alternative material to the conventional cements although they do not contain Portland cement. They can be provided by low environmental impact materials such as metakaolin, or hazardous industrial wastes and by-products such as fly-ashes. Geopolymers are obtained by a geosynthesis process involving natural or synthetic aluminosilicates in which the amorphous silicon and aluminum oxides react in strongly basic medium to form edifices which are chemically and structurally similar to natural rock (Davidovits, 1991).

The geopolymer binder synthesis follows the same reaction mechanism as the one reported by Xu and Van Deventer (2000). The reaction mixture is attacked by high pH-alkaline solution which dissolves the oxides. After amorphization of the mixture and the formation of precursors, the geopolymer precipitates in amorphous or semi-crystalline state. Previous researches on geopolymers showed that these materials are characterized by significant heat, mechanical and fire resistances (Elimbi et al., 2014) in addition to the high chemical resistance in acid medium (Bakharev, 2005a, b). Besides these benefits, geopolymers are considered as an ecological alternative to hydraulic binders for some masonry works. This is due to their low manufacturing energy consumption and their low CO_2_ emissions (Duxson et al., 2007a, b; Essaidi et al., 2013).

Various raw materials were used by researchers to synthesize geopolymers (Davidovits, 1989, 1991, 1993) and (Palomo et al., 1992; Barbosa et al., 2000; Cioffi et al., 2003; Schmücker et al., 2005). They used the metakaolin to prepare geopolymers through reaction with sodium or potassium alkaline silicates. The metakaolin is obtained by calcination of natural kaolin at temperatures higher than 600 °C. Davidovits and Davidovits (1988) burned kaolin at 500, 650, 700 and 750 °C before obtaining the geopolymer by alkaline attack. Palomo et al. (1992) studied the synthesis of geopolymer by using metakaolin that was burnt at temperatures ranging from 600 to 700 °C. Cioffi et al. (2003) investigated the effect of kaolin burning temperature (500, 550, 650 and 700 °C) on the geoploymer elaboration. Zhang and Wei (2006) calcined it at 700 °C, while Zibouche et al. (2009) have treated it at 800 °C. Chareerat et al. (2006) optimized the temperature of obtaining metakaolin which allows geopolymer binders to form. After calcination of natural kaolin at various temperatures (400, 500, 600, 700 and 800 °C) for 2, 4 and 6 hours, they concluded that the optimum calcination temperature was 600 °C. Kenne Diffo et al. (2015) optimized the calcination rate of Kaolin in order to improve the compressive strength of geopolymer, but the high value required a very low calcination speed of (1 °C/min), which is a costly process. Aboulayt et al. (2017) studied the properties of metakaolin-based geopolymers by incorporating calcium carbonate and Zaidi et al. (2017) synthesized a geopolymer from soil material, but the obtained compressive strength values were very low. Chareerat et al. (2006) synthesized a geopolymer by using a mixture of 20% of metakolin and 80% of fly-ash, while Van Jaarsveld et al. (1999) used fly-ash mixed with a small amount of kaolinite to significantly reduce the shrinkage cracks.

According to the researches carried out by Tchakoute et al. (2012), a geopolymer with mechanical properties similar to the metakaolin geopolymer can be synthesized with volcanic ash at room temperature. Thus the reactivity of volcanic ash could be modified by adding amorphous aluminosilicate materials such as metakaolin. The latter added to volcanic slag increases the amount of amorphous phase in the mixture and promotes the dissolution of alumina and silica, which leads to the formation of geopolymer binders by polycondensation reactions. This process increases the compressive strength and decreases the setting time of scorie-based geopolymer. It was concluded that volcanic slag could be used as alternative raw material in the synthesis of high compressive strength geopolymer at ambient temperature. The use of volcanic ash saves the amount of metakaolin in the synthesis of geopolymer binders.

According to another study of Tchakoute et al. (2013), the geopolymer binder obtained at room temperature from only volcanic scoria, is characterized by a low compressive strength and long setting time. Nevertheless, some of these geopolymers are expansive and can contain cracks after demolding. Other researchers have shown that geopolymers can be obtained from several sources including aluminosilicate fly ash (Van Jaarsveld et al., 1999; Xu and Van Deventer, 2000; Phair and Van Deventer, 2001; Swanepoel and Strydom, 2002; Van Jaarsveld and Van Deventer, 2002, 2003; Lee and Van Deventer, 2002a, b, 2004; Bakharev, 2005a, b; Van Deventer et al., 2007). Martin et al. (2015) studied the effect of the synthesis temperature on the compressive strength of alkaline binders (geopolymers). They investigated both alkaline cements, the first synthesized from fly ash only, and the second obtained from a mixture of fly ash and bauxite. Compression and bending mechanical tests carried out on pastes of the cements thermally treated between 25 and 60 °C have shown a brittle fracture on all specimens.

Geopolymers with Si/Al molar ratio of 2 are characterized by binding power (Davidovits, 1991; Davidovits et al., 1999; Mustafa Al Bakri et al., 2011). Ozer and Soyer-Uzun (2015) studied the development of geopolymers from metakaolin with different Si/Al molar ratios. They observed that the samples with a Si/Al ratio of 1.12 contain some crystalline components such as zeolithe A and/or sodalite. Mechanical tests related the fragility of these geopolymers to their high crystallinity, with very low compressive strength which is about 1 MPa. The X-ray diffraction analysis showed that geopolymers of Si/Al molar ratios equal to 1.77 and 2.20 had an amorphous structure which increased their compressive strength to high values between 13 and 23 MPa (Ozer and Soyer-Uzun, 2015). However, the hydrates have chemical different compositions from those of the ordinary Portland cement, which may impact the evolution of the amorphous phase more particularly, the calcium-silicate-hydrate (C–S–H) gel, which could be altered at a nanoscale, thereby affecting the mechanical properties of geopolymer (Ozçelik and White, 2016). Recent works have revealed the effect of the interfacial zones (those between crystalline and amorphous nanodomains) on the mechanical properties of geopolymers by studying various Si/Al ratios and water contents and giving model molecular structures (Koleżyński et al., 2018; Lolli et al., 2018).

Previous works led to the production of geopolymers of various characteristics, but with high fragility and low mechanical strength for most of them. The objective of this work is thus to synthesize a geopolymer binder of high mechanical performance from natural waste at very low temperature (40 °C). The hydraulic dam sludge of Tiaret in the west of Algeria was used for its chemical composition which is rich in silica and alumina and is subsequently valued in eco-material manufacturing. Geopolymers based on this dam sludge were compared to a geopolymer-based metakaolin synthesized in our laboratory from poor natural Kaolin. Both materials had not used before neither was the low cure temperature, which constitute the originality of this work. Previous studies focused on the use of industrial metakaolin obtained from pure natural Kaolin to produce geopolymers at temperature higher than 40 °C.

The structure and texture of the synthesized geopolymers and their mechanical properties were studied by using X-Ray Fluorescence (XRF), X-Ray Diffraction (XRD), Thermo-Gravimetric Analysis (TGA), Infrared Spectroscopy (FTIR), BET technique, Scanning Electronic Microscopy (SEM) and mechanical tests. For some civil engineering works, the manufacture of this geopolymer can replace the ordinary cement obtained at high temperature, while promoting natural waste. This material can be considered in specific applications, as an ecological alternative to the conventional cements which are potentially emitters of greenhouse gases including carbon dioxide (CO_2_), although Ouellet-Plamondon and Habert (2014) showed that only one part geopolymers were characterized by much lower carbon footprint levels than those of Portland cement based mixtures.

This project was carried out as part of the cooperation program Tassili 09 MDU 773 between “Laboratoire des Eco-Matériaux Fonctionnels et Nanostructurés” of the University USTO-MB, Oran- Algeria, and “Laboratoire Matériaux et Durabilité des Conctructions” de l’INSA-UPS, Toulouse- France.

## 2 Materials and methods

### 2.1 Sample testing

The chemical compositions of raw materials, raw mixtures and synthesized geopolymers were determined by X-Ray Fluorescence (XRF), using a Philips PW 1404 X spectrophotometer at the Algeria group of Lafarge Company. X-Ray Diffraction Analysis (XRD) with a Siemens D5000 diffractometer equipped with a variable slit opening and using Co Kα radiation was employed to characterize the raw materials and the synthesized geopolymers, as well as to control their hardening process. The diffraction analyses were conducted between 4 and 70° with a step of 0.02° and rate of 2.5s/step. The particle size distribution of raw materials was determined by laser granulometry (Mastersizer, 2000). Specific surface areas of raw materials and geopolymers were calculated from the sorption isotherm data using the BET method on an ASAP 2010 apparatus. Micromeritics Instrument Corp., Norcross, GA. The morphology of geopolymer crystals was examined by Scanning Electron Microscopy (Philips XL30), after covering the sample grain (≈5 mm of diameter) with graphite. The thermal analysis (TGA), performed in air on a Setaram Labsys thermoanalysor with a heating rate of 5 °C/min up to 1000 °C, was used to follow the geopolymer paste hardening. The Fourier Transformed Infra-Red (FTIR) analysis was carried out with a spectrometer "alpha bruker" equipped with ATR diamond of total attenuated reflectance. The objective was to investigate the functional group of geopolymer molecules and characterize this product to deduce its characteristics and binder nature. The mechanical test, carried out on cylindrical specimen of 20mm diameter and 20mm height, was meant to determine the compressive strength of the obtained geopolymer.

### 2.2 Raw materials

The sludge (SLD) used here is extracted from the hydraulic dam of Tiaret in the west region of Algeria. The natural kaolin (K) is obtained from the deposit of Djidjel in the east of Algeria. Both raw materials (dam sludge and natural kaolin) were dried to 105 °C in oven and then crushed to 80 μm. The crushed raw materials were burned at 800 °C for 24 hours, followed by rapid air cooling.

The chemical compositions of dam sludge (SLD) and natural Kaolin (K), determined by X-ray Fluorescence analysis (XRF), are shown in Table 1.

**Table 1:** Chemical compositions (wt%), by X-ray Fluorescence, of the used raw materials.

Raw Material	Mass percentage of oxides (Wt%)										
	SiO_2_	Al_2_O_3_	CaO	Fe_2_O_3_	MgO	K_2_O	Na_2_O	SO_3_	P_2_O_5_	TiO_2_	LOI*
K	53.14	27.72	2.63	0.75	0.40	3.77	0.20	0.36	0.17	0.88	9.88
SLD	39.6	12.70	16.46	5.92	3.20	2.65	0.05	0.54	0.17	0.56	17,95

(*) LOI: Loss Of Ignition.

### 2.3 Synthesis procedure

To synthesize a geopolymer binder of chemical formula (4SiO_2_.Al_2_O_3_.K_2_O), with Si/Al and Si/K molar ratios equal to 2, an alkaline solution was prepared by mixing an aqueous solution of potassium hydroxide (8M, 99% of KOH pallets dissolved in demineralized water) and pure alumina powder. The mixture was put in a sealed 100ml-glass-container to prevent any reaction with atmospheric CO_2_, and then heated at 75 °C for 16 hours. After cooling the liquid mixture (alkaline solution-alumina), the calcined raw material (dam sludge or kaolin) was added gradually and mixed for 10 minutes. The mixture paste so prepared was introduced into a silicone cylindrical mold, of 20mm diameter and 20mm height, and then placed on a vibrating plate for 5 minutes to drive out the trapped air bubbles. The prepared paste was covered by a thin polyethylene film and heated to 40 °C in an oven. After a heating period of 48 hours, the film was removed and the geopolymer sample was demoulded and conserved at 40 °C in the oven. Six samples of geopolymer were prepared for each synthesis to ensure the reproducibility of results and the friability of the experimental protocol.

Table 2 illustrates the chemical formulae and ICDD card numbers of minerals phases contained in the raw materials and the synthesized geopolymers.

**Table 2:** Chemical formulas and ICDD cards numbers of the found mineral phases.

Mineral Phases	Designation	Chemical Formula	ICDD no.
Quartz	Q	SiO_2_	33–1161
Kaolinite	K	Al_2_Si_2_O_5_(OH)_4_	14–164
Siderite	S	FeCO_3_	29–0696
Calcite	C	CaCO_3_	05–0586
Illite	I	K_0.7_Al_2_(Si,Al)_4_O_10_(OH)_2_	29–0370
Kalicinite	Ka	KHCO_3_	12–292
Synthetic Muscovite	M	KAl_2_Si_3_AlO_18_(OH)_2_	07–25
Kilchoanite	Ki	Ca_6_(SiO_4_) (Si_3_O_10_)	29–0370
Tobermorite	T	(Ca,Na,KH_3_O) (Si,Al)O_3_AH_2_O	20–0544
Xonotlite	X	Ca_6_Si_6_O_17_(OH)_2_	29–0379
Phillipsite	P	K_2_Ca_2_(Al,Si)_16_O_32_13.5H_2_O	26–1310
Biotite	B	K(Fe,Mg)_3_AlSi_3_O_10_(OH)_2_	02–45
Leucite	L	K(Si_2_AlO_6_)	27–24
Anorthite	A	CaAl_2_Si_2_O_8_	12–0301
Gehlenite	G	Ca_2_Al_2_SiO_7_	35–0755
Rankinite	R	Ca_3_Si_2_O_7_	23–0124

## 3 Results and discussions

### 3.1 Characterization of raw materials

**Fig. 1:**
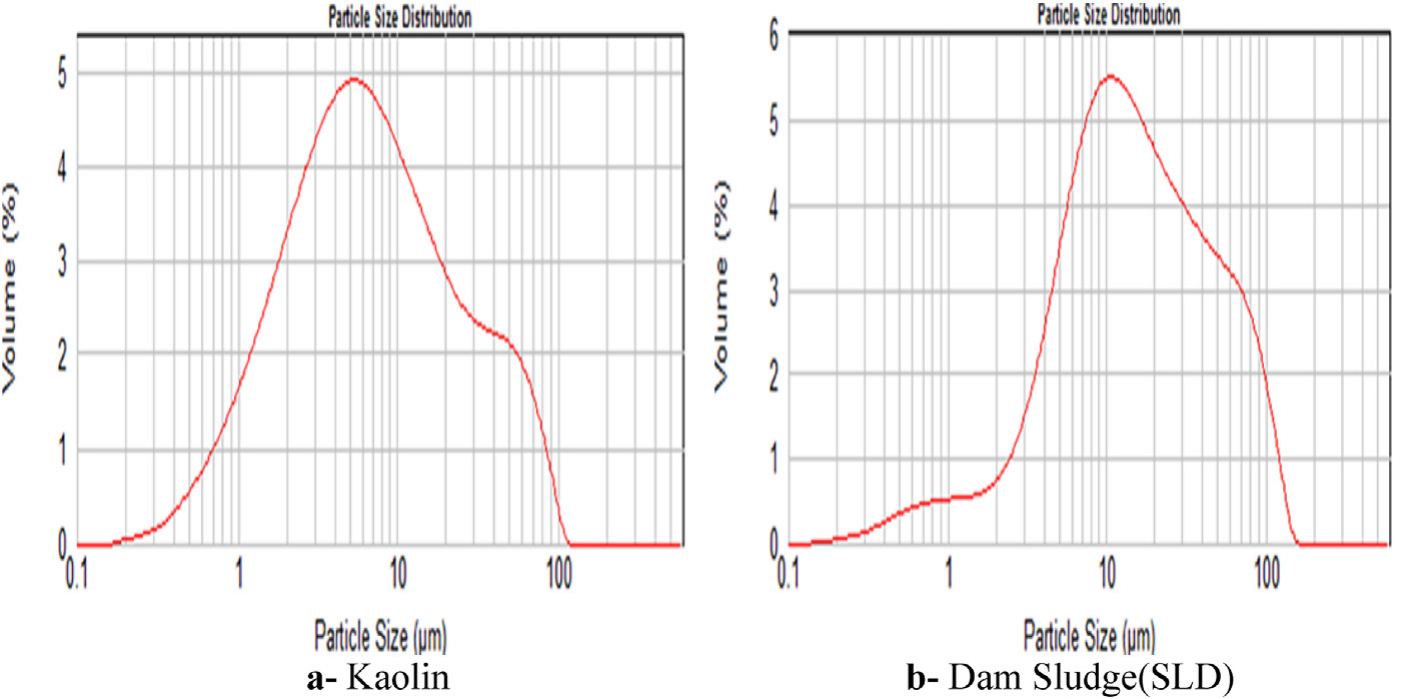
Particle size distribution of Kaolin (K) and Dam Sludge (SLD) determined by Granulometry Laser Technique.

The raw materials (dam sludge and kaolin) making up the geopolymer were characterized by the Laser Technique to show the particle granulometry of both powders. The characterization results are given in Fig. 1.

In the synthesis of solid materials, the particle size has an important effect on the reactivity of the grain surface and the chemical reaction between minerals (Diaz et al., 2010). According to Kirschner and Harmuth (2004), the raw materials of the geopolymer need a specific area between 16 and 29 m^2^/g, which is the case of Kaolin and Sludge used in this study (24 m^2^/g for Kaolin and 21 m^2^/g for SLD as it was determined by BET method). This low particle size, confirmed by the Laser technique (Fig. 1), improves the mineral activity and facilitates the geopolymerization reaction.

**Fig. 2:**
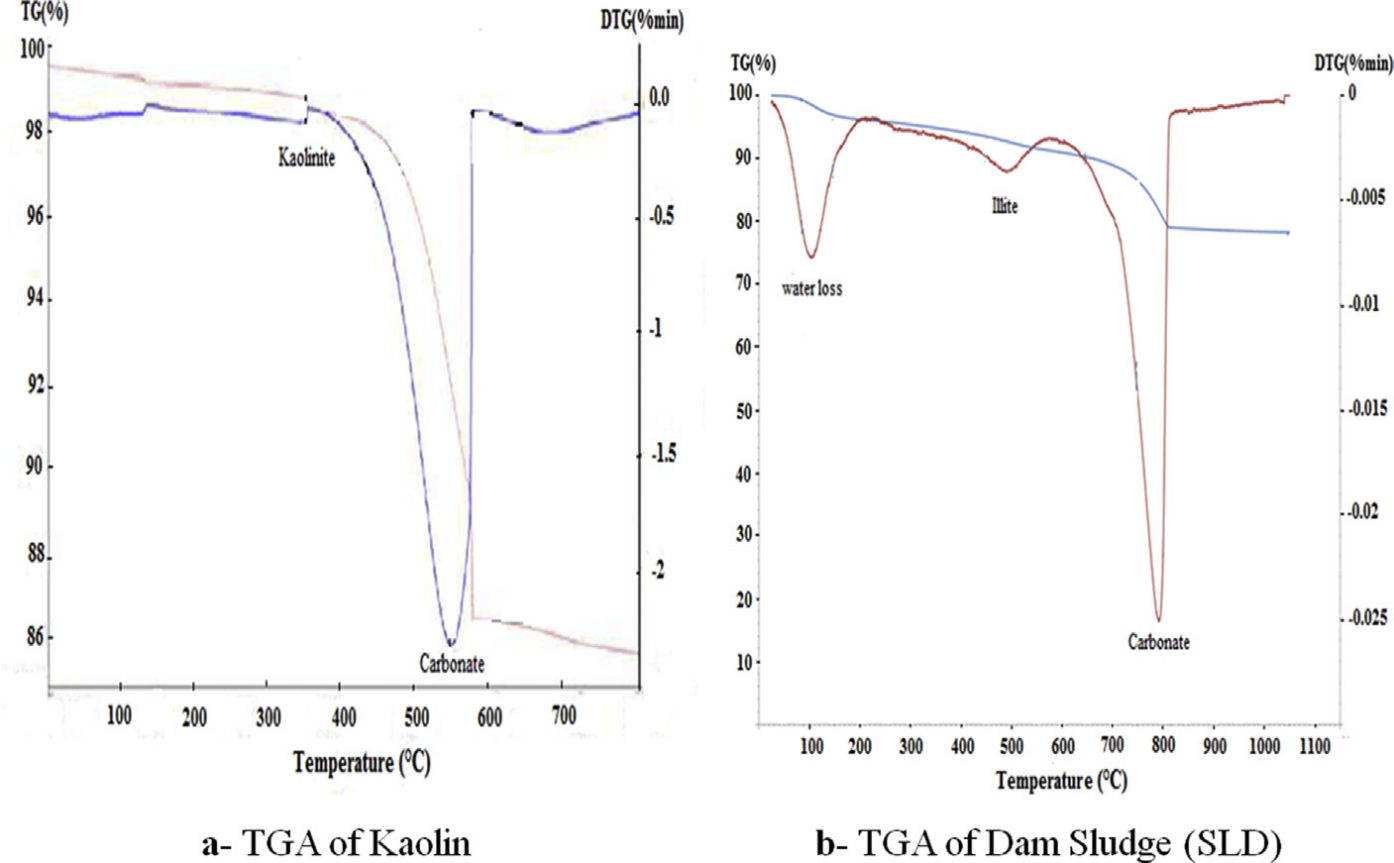
TGA-DTG Diagrams of the raw materials (Kaolin and Dam Sludge).

**Fig. 3:**
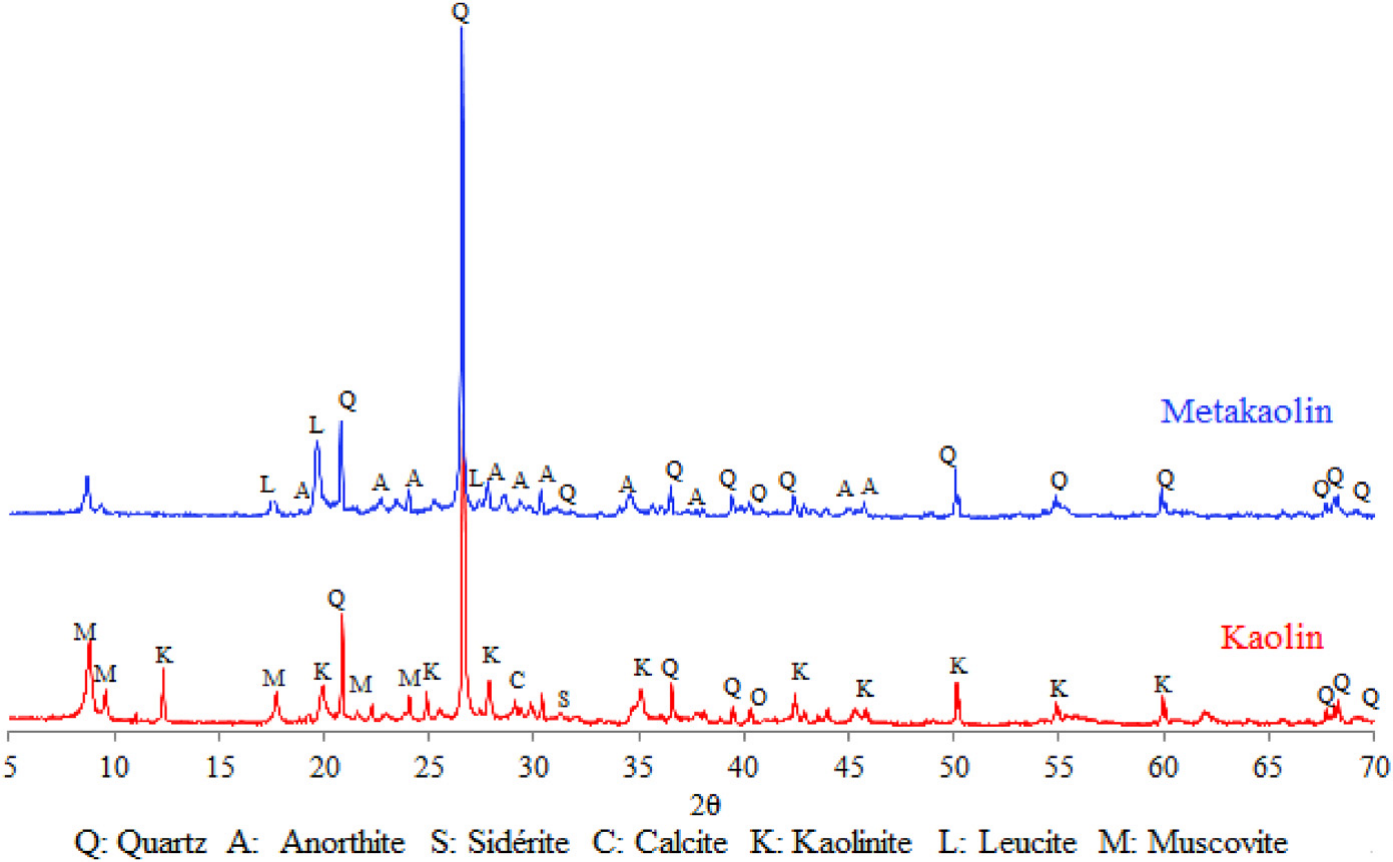
X-Ray Diffratograms of the used Kaolin (K) and Metakaolin (MK).

**Fig. 4:**
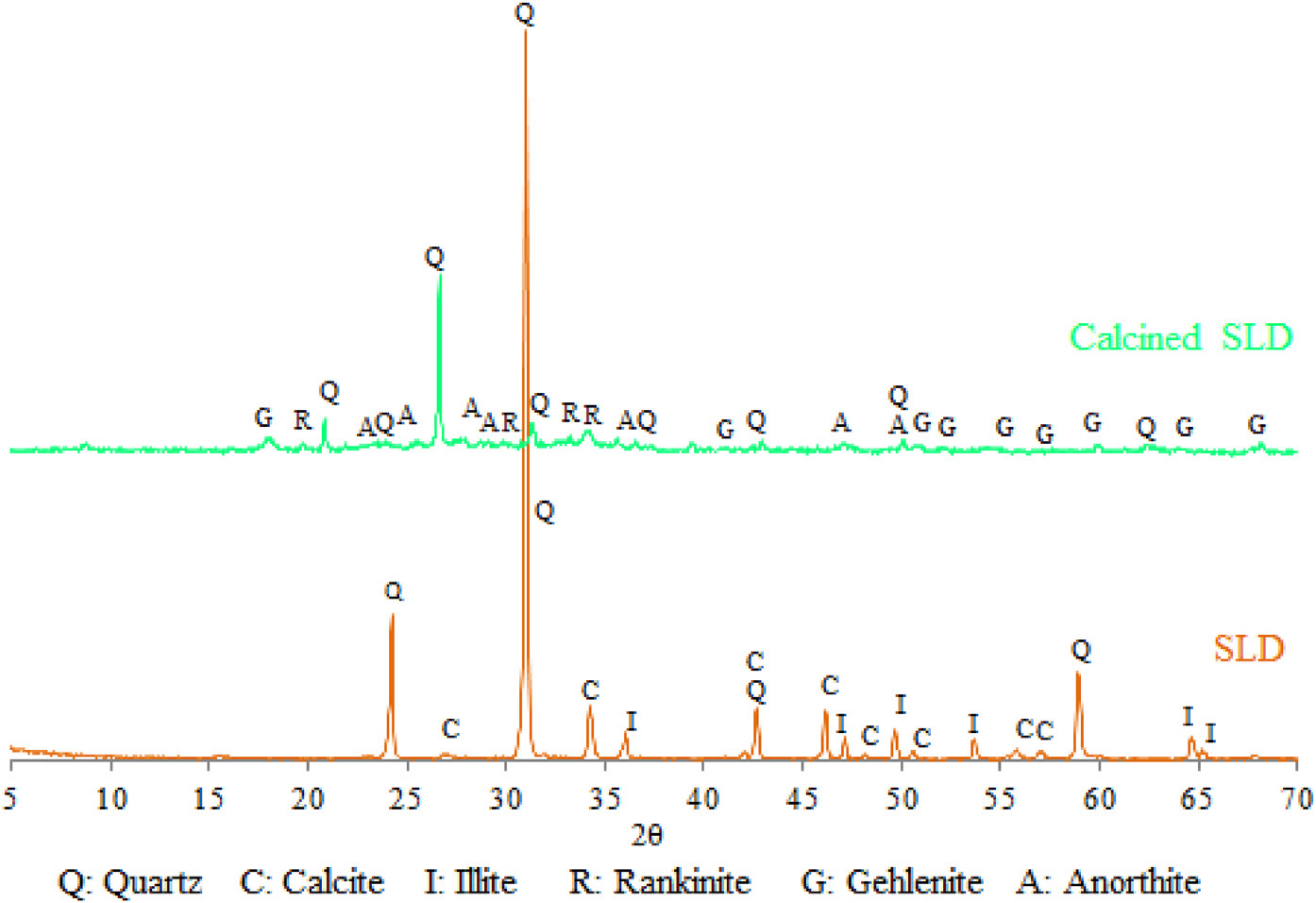
X-Ray Diffratograms of the used sludge (SLD) before and after calcination at 800 °C.

According to the thermo-gravimetric analysis (TGA), the used raw materials (Kaolin and Dam Sludge) were composed from Kaolinite, Illite and Calcium carbonate (Fig. 2). Their mineralogical compositions before and after calcination at 800 °C were determined by X-Ray Diffraction. The results are given in Figs. 3 and 4. Kaolin material contains Quartz, Kaolinite, Muscovite, Calcite and Siderite minerals (Fig. 3). After calcination at 800 °C, Kaolin was transformed into an amorphous phase, which is Metakaolin (2θ/20–35°), in addition to the formation of Anorthite after its reaction with CaO decomposed from Calcite, and other minerals like Quartz and Leucite resulting from the transformation of Muscovite (Fig. 3). Metakaolin with its disordered structure can accelerate the combination reactions conducing to the geopolymer formation. The dam sludge (SLD) used in this study is composed of Quartz, Calcite and Illite minerals (Fig. 4). After calcination at 800 °C, Calcite and Illite disappeared from the material and other minerals like Anorthite, Gehlenite and Rankinite were formed (Fig. 4). These minerals were obtained by thermal transformation and reaction between the oxides CaO, SiO_2_ and Al_2_O_3_ contained in the raw material.

**Fig. 5:**
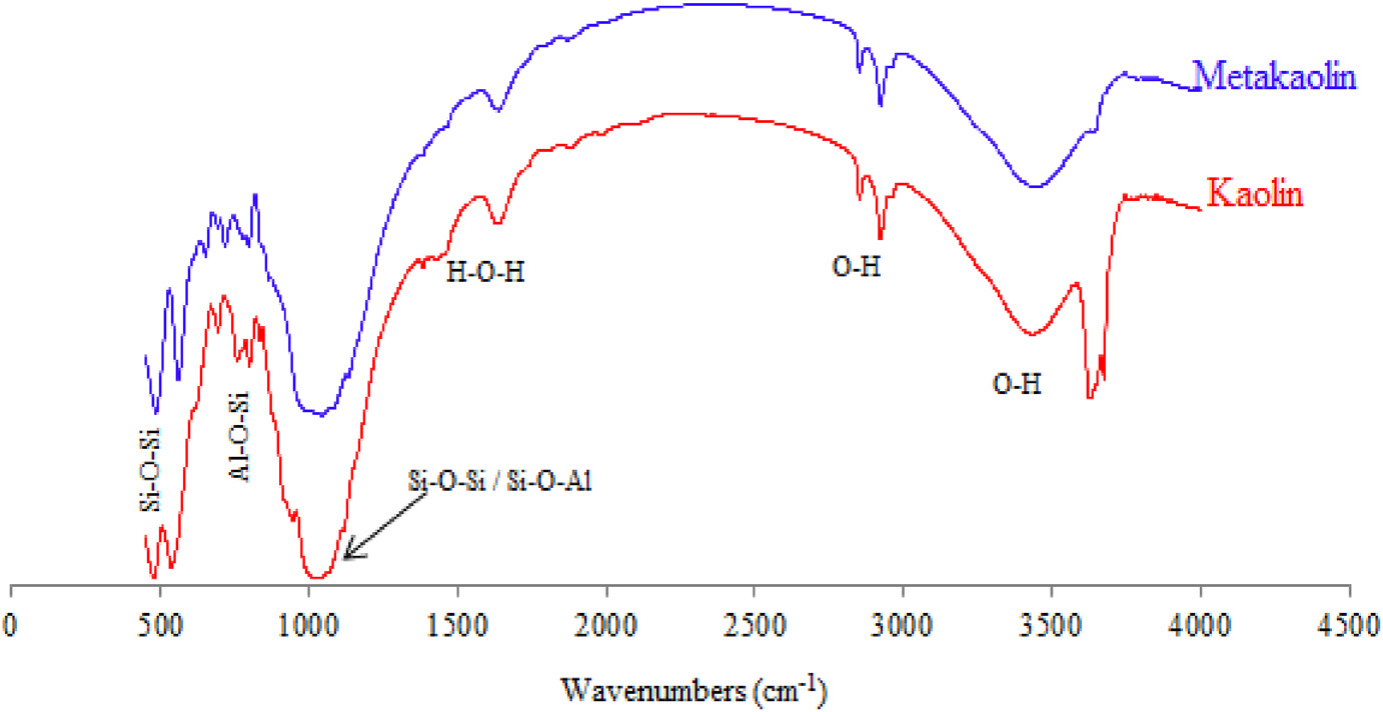
FTIR spectra of the used Kaolin and Metakaolin.

**Fig. 6:**
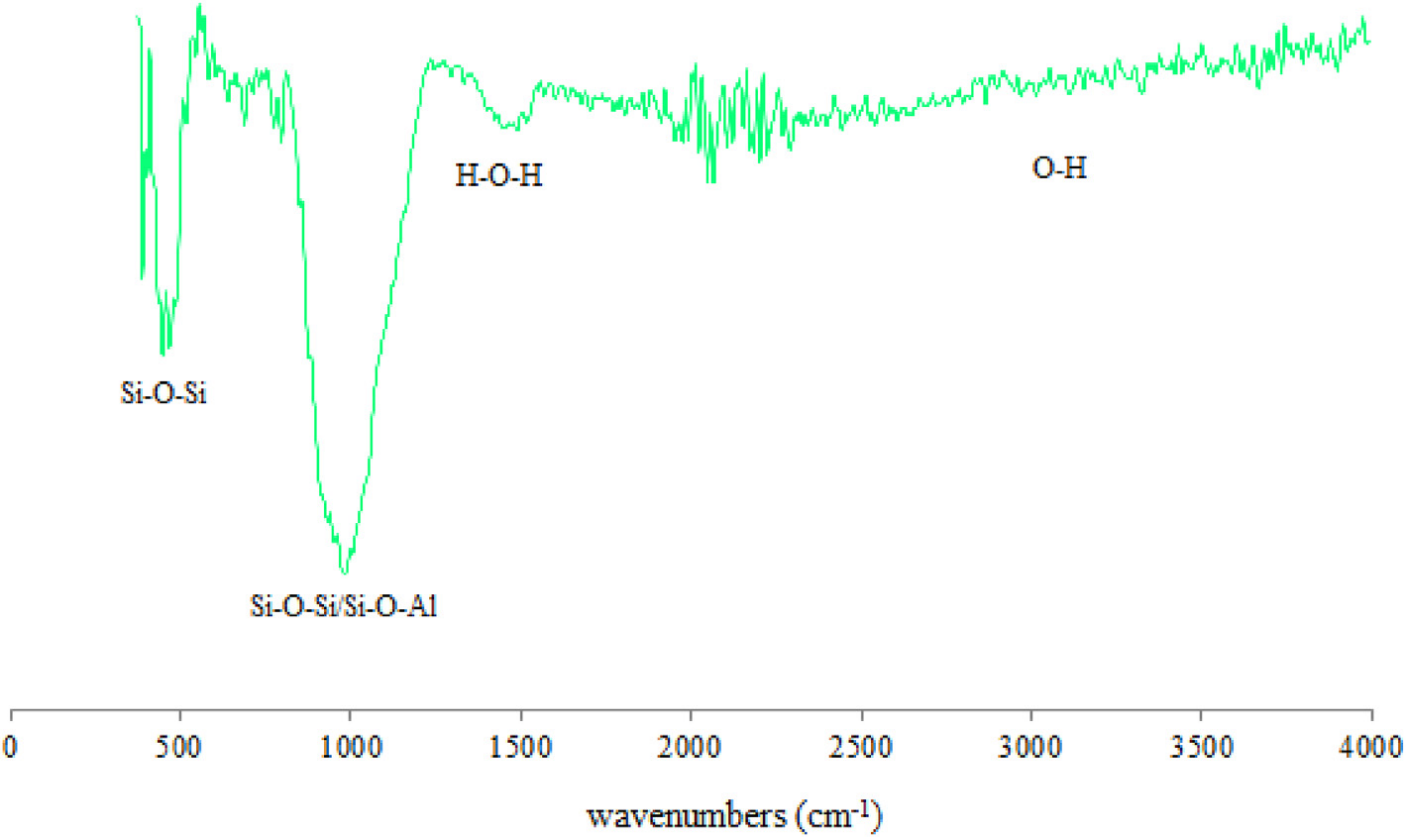
FTIR spectra of calcined sludge (SLD).

FTIR technique was also used to show the functional groups contained in Kaolin, Metakaolin and calcined sludge materials (Figs. 5 and 6). The results indicate the presence of Si–O–Al, Si–O–Si and O–H functional groups in the raw materials.

### 3.2 Chemical analysis of the synthesized geopolymers

The chemical compositions of the synthesized geopolymers (GMK and GSLD) from Kaolin and dam Sludge, determined by XRF technique, are given in Table 3. According to the chemical compositions of geopolymers, the main molar ratios were calculated and reported in Table 4.

**Table 3:** Chemical compositions of the obtained geopolymers determined by XRF technique.

Geopolymer	Mass percentage of oxides (Wt%)								
	SiO_2_	Al_2_O_3_	Fe_2_O_3_	CaO	MgO	SO_3_	K_2_O	Na_2_O	LOI*
GMK	49.61	24.56	1.18	3.37	0.96	0.04	10.16	0.02	9.75
GSLD	31.06	12.65	6.67	22.44	3.60	0.34	13.75	0.05	9.43

(*) LOI: Loss Of Ignition.

**Table 4:** Main molar ratios in the synthesized geopolymers.

Geopolymer	Molar Ratios			Mass Ratios		
	Si/Al	K/Si	K/Al	SiO_2_/Al_2_O_3_	K_2_O/SiO_2_	K_2_O/Al_2_O_3_
GMK	1.73	0.27	0.46	2.02	0.21	0.41
GSLD	2.17	0.58	1.25	2.46	0.44	1.09

**Fig. 7:**
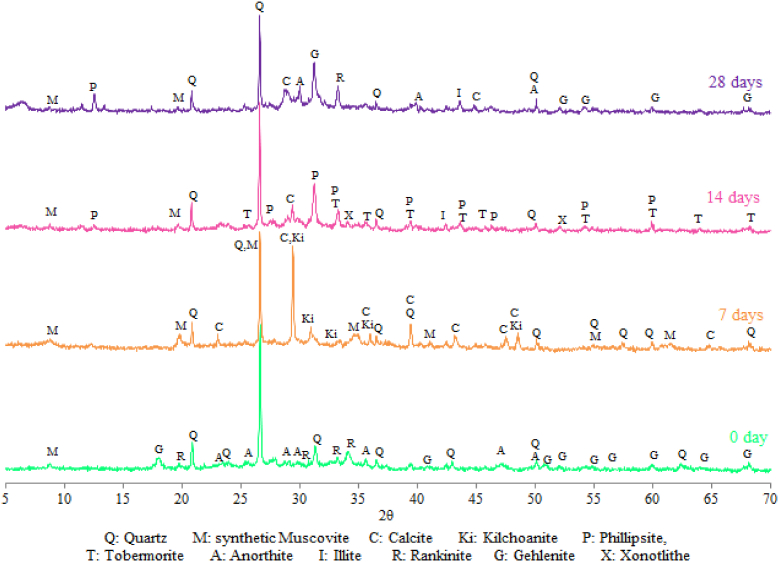
X-Ray Diffractograms of SLD Geopolymer after hardening at 0, 7, 14 and 28 days.

It was shown that the chemical composition of geopolymers (Table 3) reflects the oxide contents in the used raw materials (Table 1). Kaolin geopolymer (GMK) was rich in silica and alumina, however SLD geopolymer (GSLD) contained high percentages of calcium and iron oxides. The high presence of CaO and K_2_O in SLD geopolymer accounts for the formation of calcium and potassium aluminosilicate minerals that were found thanks XRD analysis (Fig. 7). Si/Al molar ratio was higher than 2 in GSLD and lower than 2 in the case of GMK (Table 4). All ratios (Si/Al, K/Si, K/Al) in GMK were lower than that in GSLD.

### 3.3 X-Ray Diffractograms of geopolymers after 7, 14 and 28 days of hardening

#### 3.3.1 Dam sludge geopolymer (GSLD)

The evolution of geopolymer hardening was followed by X-Ray Diffraction. Fig. 7 shows the XR Diffractogram of geopolymer obtained from dam sludge and hardened at 0, 7, 14 and 28 days. The geopolymer is composed of Quartz, Calcite, Illite, Gehlenite, Anorthite and Rankinite that were present in the raw and calcined materials, and other minerals like Muscovite, Kilchoanite, Phillipsite, Tobermorite and Xonotlite, that were formed during the geopolymerization process. The main peak intensities of minerals change with the hardening time, which is due to the formation, transformation or disappearance of mineral phases when the hardening duration increases. In the beginning of geopolymerization (before 7 days of hardening), Anorthite (CaAl_2_Si_2_O_8_), Rankinite (Ca_3_Si_2_O_7_) and Gehlenite (Ca_2_Al_2_SiO_7_) of Calcined SLD disappear from the product after turning into other minerals like Kilchoanite [(Ca_6_(SiO_4_) (Si_3_O_10_)] and synthetic Muscovite (KAl_2_Si_3_AlO_18_(OH)_2_]. This transformation is due to the presence of a high alkalinicity solution (KOH, 8M), which activates the compound crystalline structure and leads to an easy chemical reaction between different oxides to form new products (Kacimi et al., 2010; Mazouzi et al., 2014). Muscovite, Calcite, Kilchoanite, existing in geopolymer at an early age (7 days) of hardening, disappear in the long term. After 7 days, their oxides (CaO, SiO_2_, Al_2_O_3_ and K_2_O) react between themselves in water and the KOH alkaline solution to produce a mixture of calcium aluminosilicate hydrates such as Tobermorite [(Ca,Na,KH_3_O) (Si,Al)O_3_(nH_2_O)], Xonotlite [(Ca_6_Si_6_O_17_(OH)_2_] and Phillipsite [K_2_Ca_2_(Al,Si)_16_O_32_(13.5H_2_O)], which are frequently found in the Portland cement pastes. These hydrates consolidate the geopolymer texture and then increase the mechanical strength. The calcite (CaCO_3_) was detected in geopolymer after 7 days of hardening despite its non-attendance in the starting mixture (Fig. 7). This was due to the reaction of small part of CaO contained in minerals with the atmospheric CO_2_ during the geopolymerization. The presence of calcite leads to the reinforcement of mechanical strength of geopolymer (Yip et al., 2004, 2005; Buchwald et al., 2005). The formation of amorphous phase in the shape of a broad hump in XRD at 2θ/26°–38° was also observed. The unclear presence of XRD hump (Fig. 7) is due to the high peak intensity of quartz because of its high crystallization. This bump increases with the hardening time to reach high intensity at 28 days, which testifies to the great formation of the amorphous phase. The presence of this gel phase of aluminosilicate hydrates is the most important indicator of the high hardening of the geopolymer paste (Xu and Van Deventer, 2000; Komnitsas and Zaharaki, 2007; Tchakoute et al., 2012; Ozer and Soyer-Uzun, 2015).

#### 3.3.2 Kaolin geopolymer (GMK)

**Fig. 8:**
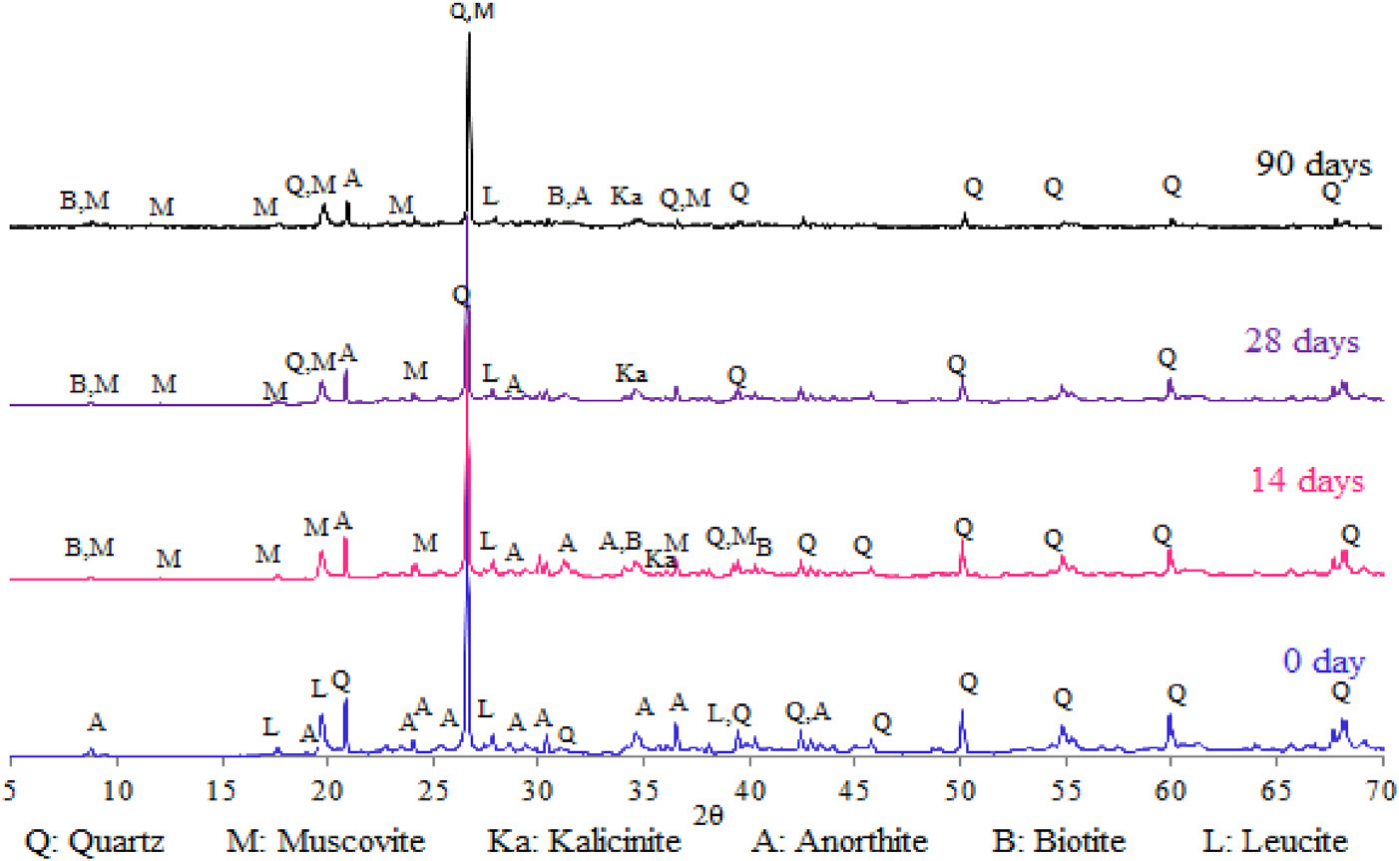
X-Ray Diffractograms of Metakaolin Geopolymer after hardening at 0, 14, 28 and 90 days.

The hardening evolution of geoplolymer obtained from kaolin is studied by XRD as shown in Fig. 8. At starting time (0 days) the geoploymer mixture contains in addition to Quartz, Anorthite (CaAl_2_Si_2_O_8_) and Leucite (KSi_2_AlO_6_) which are anhydrites of calcium and potassium aluminosilicates contained in the metakaolin. After 14 days of geopolymerization, other minerals were formed due to the chemical reaction between aluminosilicate oxides in the KOH alkaline solution. The resulting minerals are synthetic Muscovite [KAl_2_Si_3_AlO_18_(OH)_2_], Biotite [K(Fe,Mg)_3_AlSi_3_O_10_(OH)_2_)], Kalicinite (KHCO_3_). With the hardening time the mineral XRD peaks decrease to reach very low intensities at 90 days. This is probably due to the amorphisation of hydrates in the Kaolin geopolymer. The amorphous minerals of paste lead to the consolidation of texture which improves the compressive strength of the geopolymer.

### 3.4 FTIR characterization of geopolymers at 0, 7, 14 and 28 days of hardening

#### 3.4.1 Dam sludge geopolymer (GSLD)

**Fig. 9:**
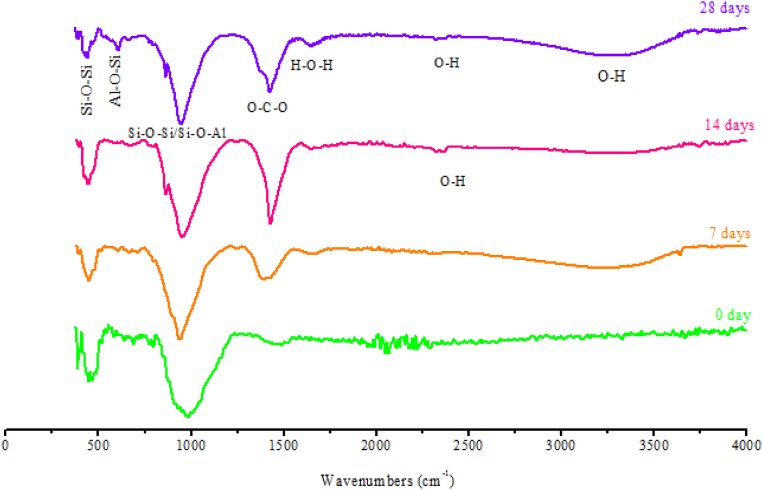
FTIR spectra of SLD geopolymer (GSLD) hardened at 0, 7, 14 and 28 days.

Fourier Transformation of Infra-Red spectroscopy (FTIR) is an analysis technique used to provide information about the vibrational transitions and rigidity of intra and intermolecular chemical bonds (Phair and Van Deventer, 2001; Socrates, 2001). FTIR spectra of hardened geopolymers at 0, 7, 14 and 28 days are given in Fig. 9. The different bands that appear in the spectra are described as follows:

- Broad band of low intensity is spread between 3000 and 3500 cm^−1^ corresponding to the stretching vibration of OH chemical bond of water molecules linked by hydrogen bonding (Panias et al., 2007). The band intensity increases with the hardening time which testifies to the bond consolidation.
- Band at 2360 cm^−1^ corresponding to the stretching vibration of OH with strong hydrogen bonding (Lee and Van Deventer, 2002a, b). This band is very weak and appears after 14 days of hardening while its intensity increases at 28 days which testifies to the consolidation of geopolymer molecules with the hardening time.
- Band at 1650 cm^−1^ is attributed to the bending vibration of vibrationnal transition of H–O–H bonds (Maragkos et al., 2009). This testifies to the presence of chemical water in pastes.
- Band at around 1430 cm^−1^ for the functional group (O–C–O) is due to the carbonation (Yousuf et al., 1993) which confirms the presence of Calcite in geopolymer, as already shown by XRD analysis (Fig. 7). The band intensity is very weak at the beginning of hardening but becomes very strong and broad with the polymerization age. This explains the formation of Calcite which causes the consolidation of the molecular texture of the hardened geopolymer (Yip et al., 2004, 2005; Buchwald et al., 2005).
- Another area contains a large band at around 950-1000 cm^−1^ near a shoulder of small intensity at 840 cm^−1^. These bands are assigned to the symmetric and asymmetric vibrations of valence bonds Si–O–Si and Si–O–Al (Gadsden, 1975; Villa et al., 2010).
- Band at 570-600 cm^−1^ for double ring structures formed by Si and Al tertrahedra (Flanigan et al., 1971). This band appears after 28 days of hardening.
- Another band at 445 cm^−1^ which was previously assigned to in-plane bending of the Si–O bonds is found inside the basic aluminosilicate tetrahedron (Ortego et al., 1991). Intensity increases with the hardening age.

The presence of all these FTIR bands shows the adequate formation of geopolymer structure. It has been shown that the intensities of all FTIR bands (Fig. 9) increase with the hardening age of geopolymer. This is due to the reinforcement of the chemical bonds of geopolymer structure which leads to the consolidation of its matrix texture and improves its compressive strength.

#### 3.4.2 Kaolin geopolymer (GMK)

**Fig. 10:**
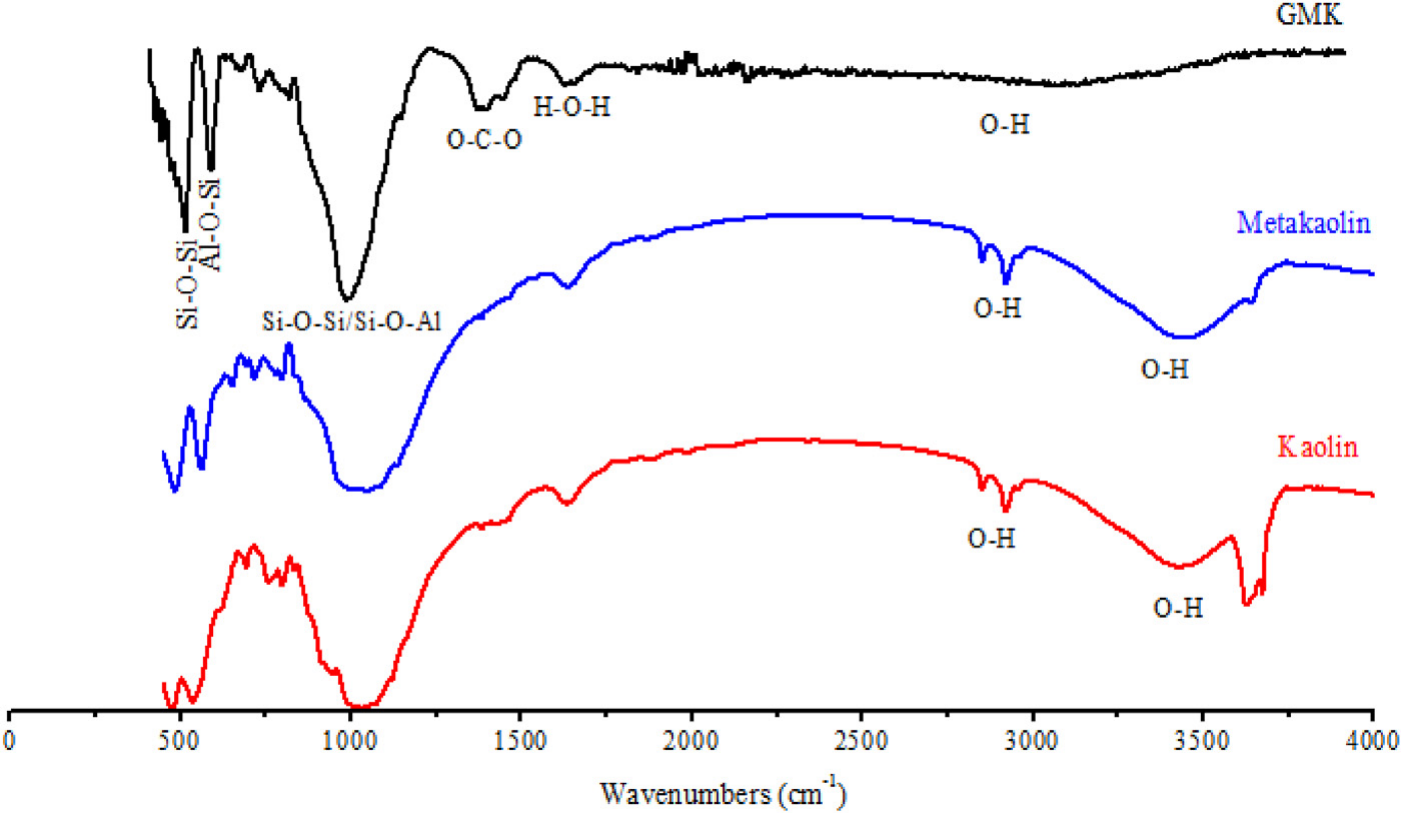
FTIR spectra of Kaolin geopolymer hardened at 28 days and its raw materials.

Fig. 10 shows the FTIR spectra of geopolymer after 28 days in addition to the used Kaolin and Metakaolin. The Kaolin spectrum is characterized by a double band of O–H between 3550 and 3700 cm^−1^ which explain the presence of chemical water in the Kaolinite structure. After calcination at 800 °C these bands disappear, which confirms the formation of metakaolin. Another band at between 2800 and 3600 cm^−1^ is also attributed to the O–H of low energy bond resulting from the humidity and adsorbed water which disappears from geopolymer. At around 1630 cm^−1^ there is a band of water functional group (H–O–H) in Kaolin, Metakaolin and Geopolymer. The band at between 1300 and 1430 cm^−1^ in geopolymer product is attributed to O–C–O functional group resulting from the carbonation (Yousuf et al., 1993) which produces the Kalicinite (KHCO_3_) already observed in X-Ray Diffractograms (Fig. 6). It has also been shown that the intensities of the bands characterizing the functional groups Si–O–Si (550 cm^−1^) and Si–O–Al (620 cm^−1^) decrease when the Kaolin was calcined, which accounts for the destruction of the bonds Si–O and Al–O of clay. The band at between 900 and 1100 cm^−1^ is very large in Kaolin and Metakaolin, but in geopolymer it becomes very sharp and more intense which explains the very strong bond of aluminosilicate molecules in geopolymer resulting in the increase of its mechanical strength. This characteristic is also revealed by the appearance of an intense and sharp band in the geopolymer spectrum for Si–O–Al at 620 cm^−1^.

### 3.5 Thermal analysis of geopolymers

**Fig. 11:**
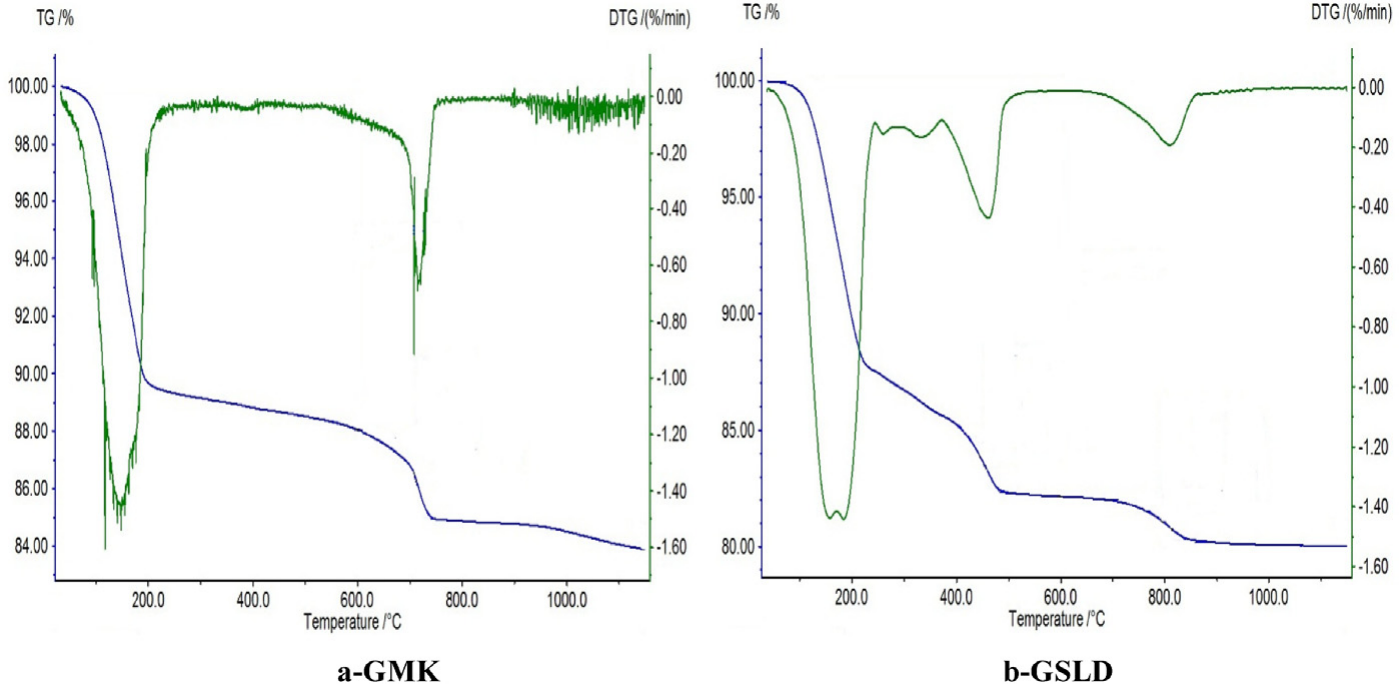
TGA-DTG Diagrams of Kaolin and Sludge Geopolymers hardened at 28 days.

The synthesized geopolymers from dam Sludge (SLD) and Kaolin (K) were characterized by Thermo-Gravimetric Analysis (TGA). The results are given in Fig. 11.

For the Kaolin Geopolymer (GMK) (Fig. 11-a), the TG Diagram shows a peak between 100 and 200 °C which is attributed to the presence of aluminosilicate hydrates (synthetic Muscovite [KAl_2_Si_3_AlO_18_(OH)_2_]) and Biotite [K(Fe,Mg)_3_AlSi_3_O_10_(OH)_2_)]) contained in the geopolymer pastes according to DRX analysis (Fig. 8). Another peak is located at 720 °C revealing the presence of carbonate in geopolymer pastes due the formation of Kalicinite compound (KHCO_3_). This compound formation, detected also by XRD and FTIR, leads to the improvement of the hardening of geopolymer.

TGA of SLD geopolymer (GSLD) (Fig. 11-b) shows a double peak at between 100 and 200 °C attributed to the hydrates (calcium aluminosilicate hydrates) with zeolitic water. The peaks at between 300 and 400 °C explain the presence of chemical water bonded with aluminum and silicon, and the peak at 450 °C is attributed to the calcium hydrate contained in the geopolymer structure after transformation of CaCO_3_ (Jouenne, 1984). The peak at around 800 °C testifies to the presence of carbonates like Calcite (CaCO_3_), which is in accordance with XRD and FTIR analyses (Figs. 7 and 9).

TGA analysis of Kaolin and SLD geopolymers confirms the mineralogy found in the products by XRD and FTIR techniques resulting in their hard structure and texture.

### 3.6 Scanning electronic microscopy (SEM) study of the synthesized geopolymers

**Fig. 12:**
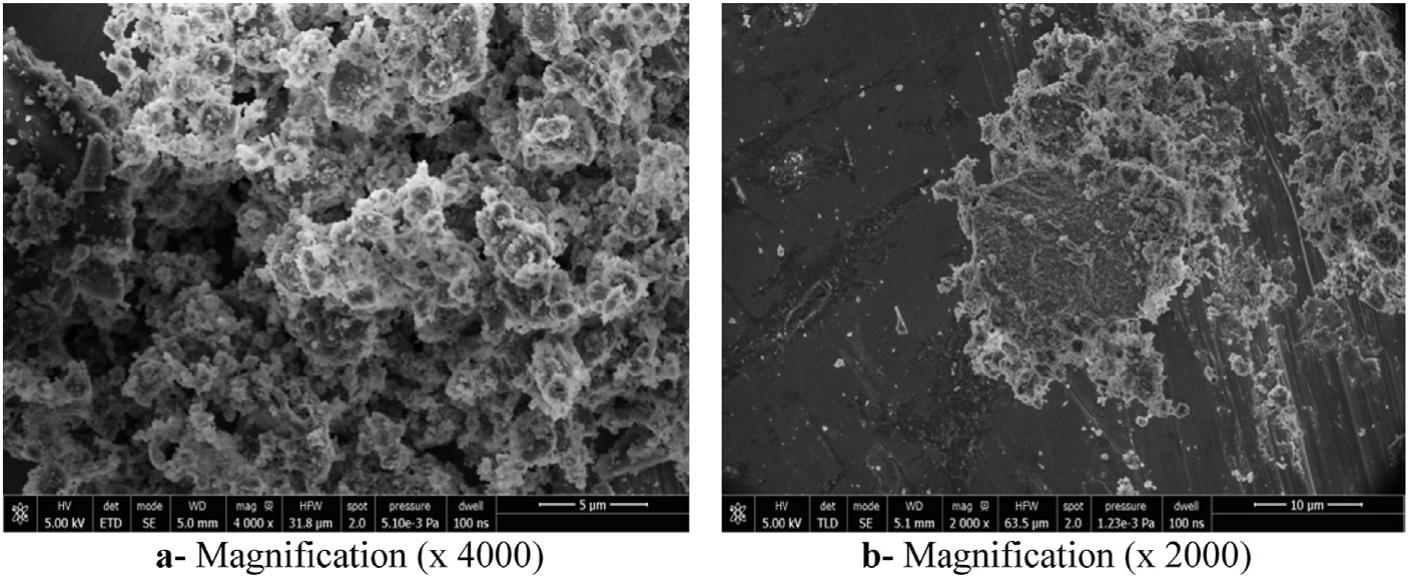
SEM micrographs of Metakaolin Geopolymer (GMK).

**Fig. 13:**
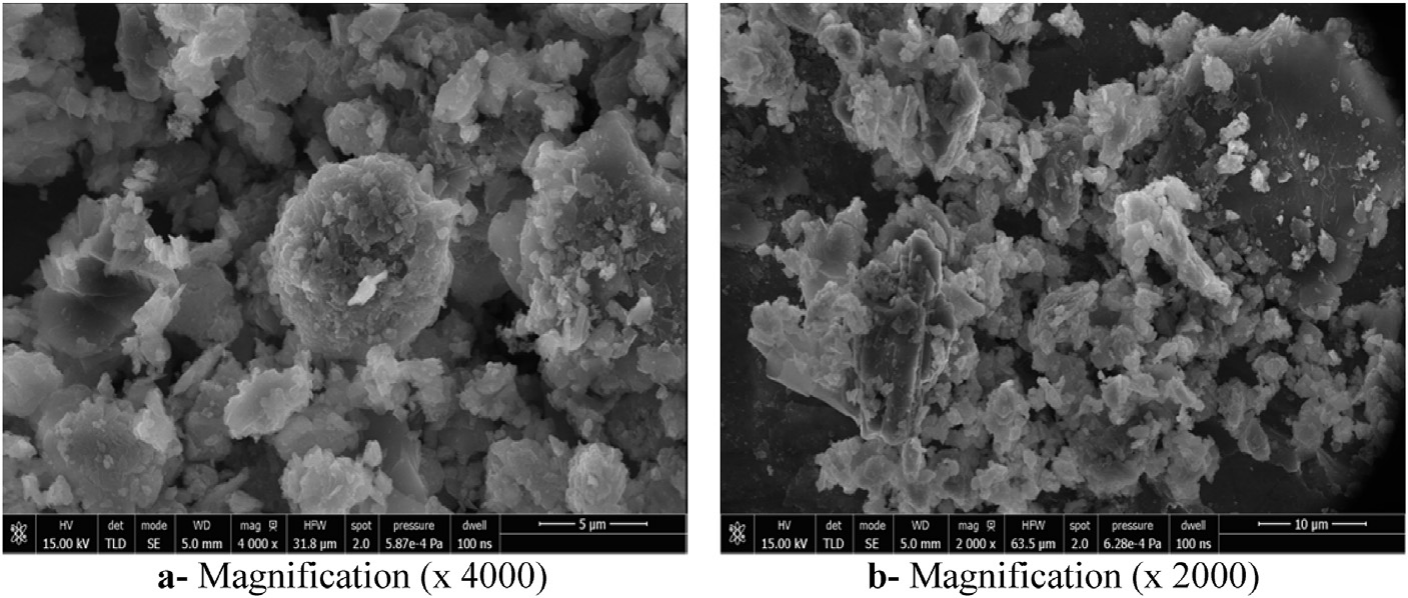
SEM micrographs of Sludge Geopolymer (GSLD).

The SEM technique was used to show the morphology and size of crystal and amorphous phases, and the porosity of the Kaolin and Sludge Geopolymers. The SEM micrographs are given in Figs. 12 and 13.

SEM micrographs (Figs. 12 and 13) indicate that geopolymers obtained from Kaolin (MK) and Sludge (SLD) have been characterized by an organized compilation of homogeneously distributed grains. They also indicate the presence of undissolved particles near crystal and amorphous phases of geopolymer. This is due to the incomplete polycondensation of raw particles because of the high potassium content in the used alkaline solution (8M), which inhibits the formation of aluminoslicate gel (Maragkos et al., 2009). However, the texture of the product shows the presence of some micropores which is a characteristic of geopolymers. It was observed that SLD the geopolymer paste is less compact than that of the Kaolin geopolymer, which is in agreement with BET analysis giving 10.2 m^2^/g for GMK and 11.8 m^2^/g for GSLD. This difference is due to the amorphous phase amount that is more important in the case of Kaolin geopolymer.

### 3.7 Compressive strength of the synthesized geopolymers

**Fig. 14:**
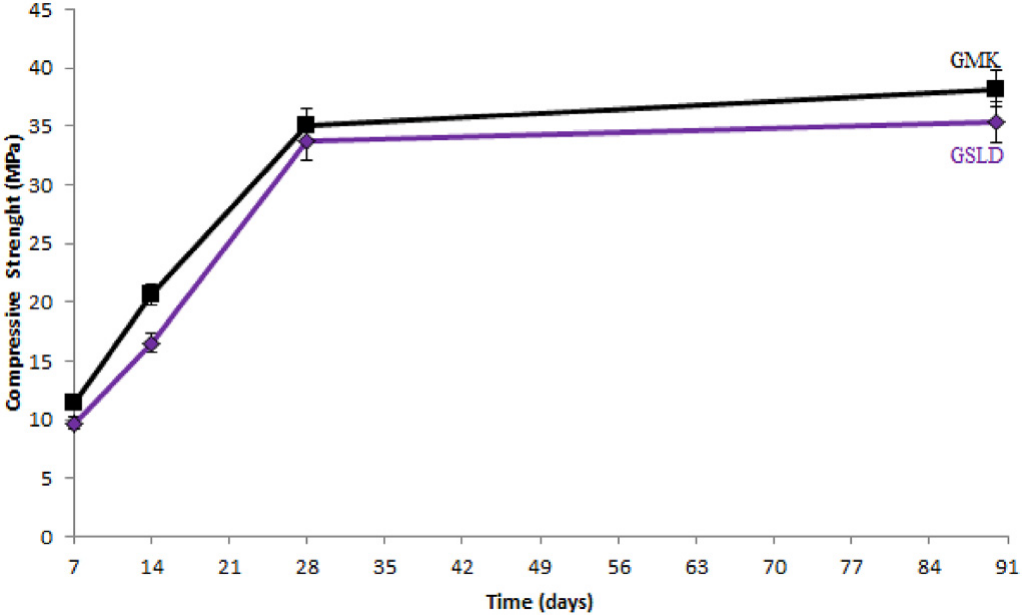
Compressive strength evolution of Kaolin and Sludge geopolymers.

The obtained geopolymers are intended for a binder application, so it is necessary to know their hydration activity and their mechanical properties. To do this, the compressive strength after 7, 14, 28 and 90 days of hardening of geopolymer pastes was measured at room temperature using an electro-hydraulic press. The compressive strength tests were carried out on six cylindrical specimens (20 mm diameter, 20 mm height) for each age after a process of polishing to obtain flat and parallel surfaces. The results are illustrated in Fig. 14.

As it was shown by many researchers, the compressive strength of geopolymers depends on several parameters: undissolved Si–Al particles (Xu and Van Deventer, 2000), gel and crystal phase amounts (Lee and Van Deventer, 2002a, b), particle size distribution (Kumar et al., 2005; Wang et al., 2005), CaO and K_2_O amounts (Van Jaarsveld et al., 1998; Phair and Van Deventer, 2001; Lee and Van Deventer, 2002a, b; Xu and Van Deventer, 2002; Van Jaarsveld and Van Deventer, 2003; Yip et al., 2004, 2005; Buchwald et al., 2005; Minarıkova and Skvara, 2005; Dombrowski et al., 2007), Si/Al and K/Al molar ratios (Davidovits et al., 1999; Duxson et al., 2005; De Silva et al., 2007; Khale and Chaudhary, 2007), KOH concentration (Swanepoel et al., 1999; Phair and Van Deventer, 2001; Wang et al., 2005), curing temperature (Khalil and Merz, 1994; Van Jaarsveld and Van Deventer, 2002; Bakharev, 2005a, b; Miller et al., 2005; Duxson et al., 2007a, b), texture and porosity of geopolymer paste (Kumar et al., 2005; Duxson et al., 2007a, b).

Fig. 14 shows that the compressive strength of geopolymer pastes increases with the hardening time to achieve high values at 28 days: 33.73 and 35.12 MPa for Sludge and Kaolin geopolymers successively. After 28 days the evolution of hardening was very slow, of which the compressive strength at 90 days was 35.38 MPa for SLD geopolymer (GSLD) and 38.25 MPa for Kaolin geopolymer (GMK). This explains the fast hardening of geopolymer at an early age. The hardening evolution of geopolymer pastes was due to the chemical reactions of aluminosilicates contained in Metakaolin and calcined Sludge with alkalis (K_2_O) resulting from the used alkaline solution (KOH).

The mechanical performances are related to the mineralogical composition, the structural and textual characteristics and the bonding nature of the obtained geopolymers. As it was shown by XRD analysis (Figs. 7 and 8), the hardening products are rich in amorphous and structural minerals of calcium aluminosilicate hydrates in the system CaO–SiO_2_–H_2_O, CaO–Al_2_O_3_–H_2_O and CaO–SiO_2_–Al_2_O_3_–H_2_O similar to those found in conventional cement pastes such as Tobermorite and Xonotlite, in addition to others (synthetic Muscovite, Phillipsite, Biotite) containing Ca, K, Na and Mg strongly linked to Si and Al by oxygen bridges with chemical bonds. These minerals are characterized by a low microporosity and very hard bonds Si–O, Al–O, Si–O–Al, Si–OH, Al–OH, Si–O–Al–OH, Ca–O–Si, K–O–Si–OH, Na–Al–OH, which were shown by the FTIR analysis (Figs. 8 and 9). These characteristics, in addition to the compact texture shown by SEM micrographs (Figs. 12 and 13), are responsible for the important compressive strength of the obtained geopolymers. According to Duxson et al. (2005) and Ozer and Soyer-Uzun (2015), the compressive strength of Metakaolin Geopolymer gains its maximum value when Si/Al and K/Al molar ratios achieve 1.7 to 2.2 which is in agreement with our work in the case of Si/Al ratio, but the K/Al ratio was less than this value especially in Kaolin geopolymer. Khale and Chaudhary (2007) have also proven that the highest compressive strength will be obtained when K_2_O/SiO_2_ and K_2_O/Al_2_O_3_ ratios achieve 0.2–0.48 and 0.8–1.6 respectively, which is in accordance with our work for the case of SLD. However, GMK have presented lower K_2_O/Al_2_O_3_ ratio. For the same authors, SiO_2_/Al_2_O_3_ mass ratio must be between 3.3 and 4.5 to obtain hard geopolymers. Our results allow us to deduce that the compressive strength values of both synthesized geopolymers were lower than what would be expected if the concentration of KOH and SiO_2_ amount were higher. The compressive strength of Kaolin geopolymer (GMK) is better than that of Sludge geopolymer (GSLD). This is due to the texture of the paste which is more compact in GMK according to the SEM study (Figs. 12 and 13). The high percentage of CaO in SLD geopolymer has led to its mechanical reinforcement by producing some calcium aluminosilicates of binder character similar to that contained in cement pastes. This is supported by several researches (Van Jaarsveld et al., 1998; Phair and Van Deventer, 2001; Lee and Van Deventer, 2002a, b; Xu and Van Deventer, 2002; Yip et al., 2004, 2005; Buchwald et al., 2005; Minarıkova and Skvara, 2005; Dombrowski et al., 2007).

## 4 Conclusion

Geopolymers with adequate mechanical performance were obtained from poor quality Algerian Kaolin and dam Sludge. The compressive strength values of the obtained geopolymers are similar to those of geopolymers synthesized by other researchers. The following conclusions can be drawn from this study:

1 The dam Sludge geopolymer is composed of a mixture of calcium aluminosilicate hydrates: Tobermorite, Xonotlite, Phillipsite and amorphous phase. Muscovite, Biotite, Kalicinite and amorphous phase are contained in the Kaolin geopolymer. All these hydrates consolidate the texture of the geopolymers and then increase the mechanical strength.
2 FTIR analysis of both geopolymers of Kaolin and dam Sludge show the presence of several bands, Si–O, Al–O, Si–O–Al, Si–OH, Al–OH, O–H, which could testify to the formation of the aluminosilicates hydrates. The increase of the band intensities in proportion of the hardening time seems to explain the development of the bond strength leading to the structure strengthening of geopolymer.
3 TGA diagrams of Kaolin and Sludge geopolymers confirm the presence of aluminosilicate hydrates in crystal or amorphous state, that were detected by XRD and FTIR analysis. These hydrates lead to the reinforcement of the texture of the geopolymers.
4 The SEM observations have shown a compact texture with some micropores and undissolved particles. An amorphous phase including crystal phases has also been observed. The geopolymer compactness was confirmed by BET measurements giving specific surface values ranging from 10 to 12 m^2^/g.
5 The mineralogical characteristics of the synthesized geopolymers in addition to their structural and textural properties, have led to a rapid development of their compressive strength. The mechanical strength values are considered acceptable compared to those found in the literature. The results enabled us to classify the obtained geopolymers as binder materials equivalent to cement of class 32.5. A modification in K/Al molar ratio to be near 2 could increase the compressive strength value to achieve its maximum. These mechanical performances allow geopolymers to be applied in the construction field as ecological binder.

## Declarations

### Author contribution statement

Larbi Kacimi: Conceived and designed the experiments; Wrote the paper.

Meriem Merabtene: Performed the experiments.

Pierre Clastres: Analyzed and interpreted the data; contributed reagents, materials, analysis tools or data.

### Funding statement

This research did not receive any specific grant from funding agencies in the public, commercial, or not-for-profit sectors.

### Competing interest statement

The authors declare no conflict of interest.

### Additional information

No additional information is available for this paper.
